# *SDHC* methylation in gastrointestinal stromal tumors (GIST): a case report

**DOI:** 10.1186/s12881-015-0233-7

**Published:** 2015-09-28

**Authors:** Milena Urbini, Annalisa Astolfi, Valentina Indio, Michael C. Heinrich, Christopher L. Corless, Margherita Nannini, Gloria Ravegnini, Guido Biasco, Maria A. Pantaleo

**Affiliations:** “Giorgio Prodi” Cancer Research Center, University of Bologna, via Massarenti 11, 40138 Bologna, Italy; VA Portland Health Care System and OHSU Knight Cancer Institute, and Division of Hematology and Oncology, Oregon Health & Science University, Portland, Oregon USA; Department of Pathology and OHSU Knight Cancer Institute, Oregon Health & Science University, Portland, Oregon USA; Department of Specialized, Experimental and Diagnostic Medicine, Sant’Orsola-Malpighi Hospital, University of Bologna, Bologna, Italy; Department of Pharmacy and Biotechnology, FaBit, University of Bologna, Bologna, Italy

**Keywords:** SDHC, Methylation, Hypermethylation, GIST, dSDH GIST

## Abstract

**Background:**

Gastrointestinal stromal tumors (GIST) recently have been recognized as a genetically and biologically heterogeneous disease. In addition to *KIT* or *PDGFRA* mutated GIST, mutational inactivation of succinate dehydrogenase (SDH) subunits has been detected in the *KIT*/*PDGFRA* wild-type subgroup, referred to as SDH deficient (dSDH). Even though most dSDH GIST harbor mutations in SDHx subunit genes, some are SDHx wild type.

Epigenetic regulation by DNA methylation of CpG islands recently has been found to be an alternative mechanism underlying the lack of SDH complex in GIST.

**Case presentation:**

We report a particular case of dSDH GIST, previously analyzed with microarrays and next-generation sequencing, for which no molecular pathogenetic events have been identified. Gene expression analysis showed remarkable down-modulation of *SDHC* mRNA with respect to all other GIST samples, both *SDHA*-mutant and *KIT*/*PDGFRA*-mutant GIST. By a bisulfite methylation assay targeted to 2 *SDHC* CpG islands, we detected hypermethylation of the *SDHC* promoter.

**Conclusion:**

Herein we report an additional case of dSDH GIST without SDHx mutation but harboring hypermethylation in the *SDHC* promoter, thus confirming the complexity of the molecular background of this subtype of GIST.

**Electronic supplementary material:**

The online version of this article (doi:10.1186/s12881-015-0233-7) contains supplementary material, which is available to authorized users.

## Background

Gastrointestinal stromal tumors (GIST) recently have been recognized as a genetically and biologically heterogeneous disease [1]. In addition to *KIT*- or *PDGFRA*-mutated GIST, mutational inactivation of succinate dehydrogenase (SDH) subunits has been detected in the *KIT*/*PDGFRA* wild-type subgroup. This latter type of GIST can be identified by negative staining of *SDHB* proteins and is defined as SDH-deficient (dSDH) GIST [2, 3].

Epigenetic regulation by DNA methylation of CpG islands has been shown to play a relevant role in the pathogenesis of several cancers. The hypermethylation phenotype has been detected in dSDH GIST, and more recently evidence of the involvement of methylation of *SDHC* in this subgroup has been reported [4–7].

Herein we reported the molecular characterization of 1 case of dSDH GIST—analyzed with microarrays, next-generation sequencing, and bisulfite sequencing—identifying, as unique molecular pathogenic event, methylation of the *SDHC* promoter.

## Case presentation

A 25-year-old woman had multifocal, epithelioid gastric GIST (CARE checklist is available as Additional file 1). *KIT* (exons 9, 11, 13, and 17) and *PDGFRA* (exons 12, 14, and 18) molecular analysis did not show evidence of mutations. The clinical/pathological features matched well with those describing dSDH GIST (female sex, young age, and multifocal and epithelioid gastric GIST). Moreover, in a gene expression analysis previously reported (GSE20710), we showed that the expression profile of this tumor (GIST_21) was similar to the profile of 3 patients (GIST_07, GIST_10, and GIST_24) who we subsequently identified as having mutated *SDHA* [3, 8]. To better characterize GIST_21, all SDHx subunits were sequenced by the Sanger method, but the analysis did not show any mutations.

To search for alterations that might not have been detected by Sanger sequencing, we performed paired-end whole exome sequencing of GIST_21 (see Additional file 2 for materials and methods), without finding any mutations in any SDHx coding sequences or flanking intronic regions. Exhaustive exome sequencing analysis revealed no pathogenic mutations in other GIST-related genes, such *PDGFRA*, *BRAF*, and neurofibromin 1, and only a nonsynonymous missense variant in exon 18 of *KIT*. This variant is described in the COSMIC database (COSM133780) in 1 case of malignant melanoma. Because the matched normal sample for this patient was not available, we were not able to determine if this *KIT* variant was somatic or germinal.

**Fig. 1:**
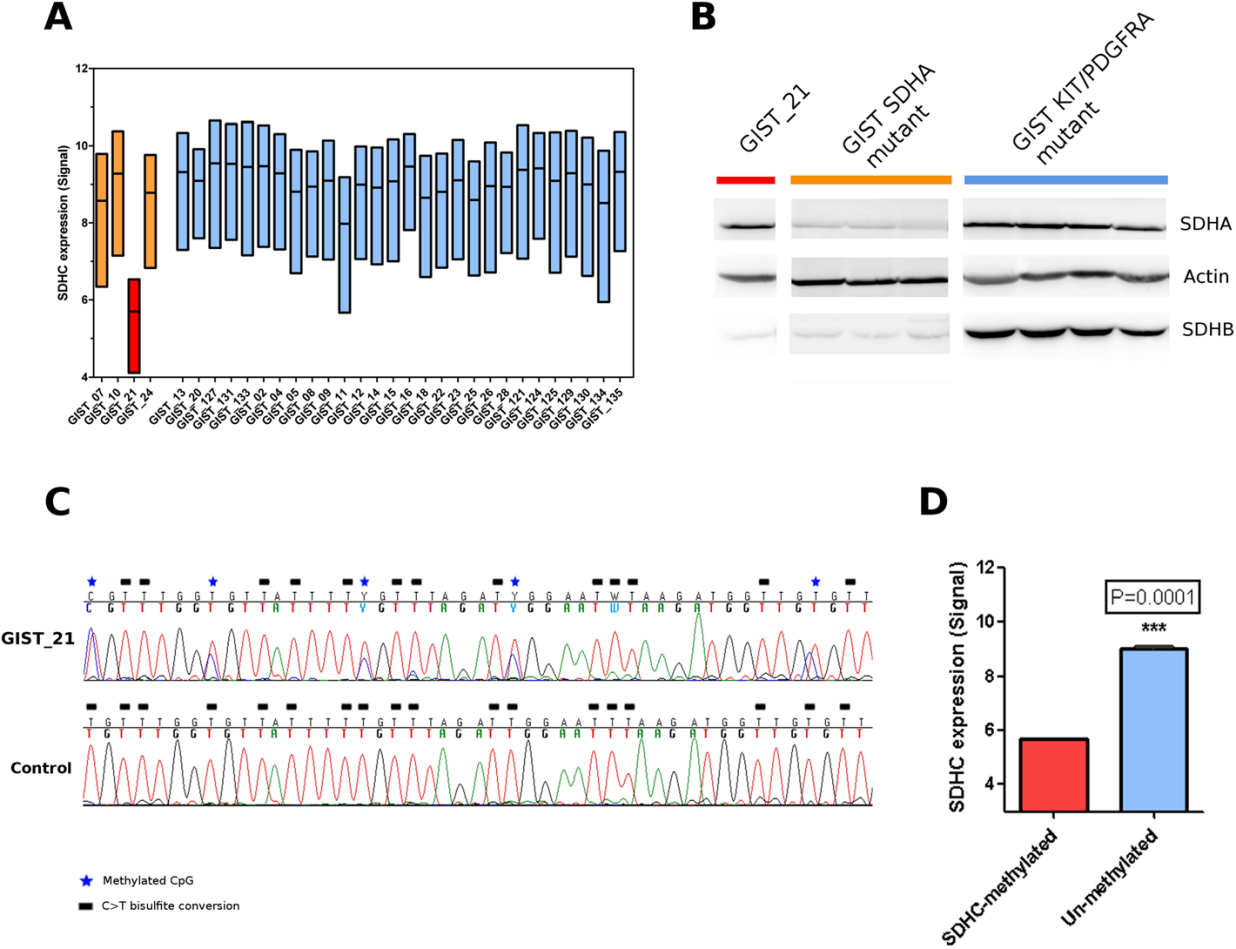
*SDHC* alteration of GIST_21. **a** Down-modulation of *SDHC* mRNA in GIST_21 (*red*) with respect to the other *SDHA*-mutated (*orange*) and *KIT/PDGFRA*-mutated (*light blue*) GIST. **b** Western blot immunostaining of *SDHA* and *SDHB* proteins. GIST_21 showed negative *SDHB *staining similar to *SDHA*-mutant GIST. Actin was used as loading control. **c** Partial chromatogram of PCR products of the promoter region of the *SDHC* gene. Tumor genomic DNA from GIST_21 and 11 GIST control samples (3 *SDHA* and 8 *KIT/PDGFRA* mutants) were treated with bisulfite before amplification and sequencing. The upper sequence, belonging to GIST_21, carries methylated CpG in heterozygous status, whereas the lower sequence is an example of methylation-negative control samples. Stars show the location of CpG dinucleotides methylated in the predicted CpG island, while boxes show the C outside of the CpG island completely converted by bisulfite to T. **d** *SDHC* mRNA expression level comparison between the methylated case (GIST_21) and the 11 methylation-negative samples (3 *SDHA* and 8 *KIT/PDGFRA* mutants). *P* value was estimated with one sample *t* test

By gene expression analysis, we examined the expression levels of the 4 subunits of the SDH complex; as shown in Fig. 1a, we detected a remarkable down-modulation of *SDHC* mRNA in GIST_21 with respect to all other GIST samples, both *SDHA*-mutant and *KIT*/*PDGFRA*-mutant GIST. Moreover, Western blot immunoassay showed that GIST_21 had normal *SDHA* but markedly decreased *SDHB* protein expression (Fig. 1b) and would be predicted to have a loss of SDH complex activity. Additionally, SNP6.0 genotyping on GIST_21 showed a normal karyotype, with no detectable loss of heterozygosity or chromosome 1q deletion [9].

Hypermethylation of the *SDHC* promoter region was recently associated with Carney triad–related GIST and dSDH GIST without SDHx mutation [6, 7]. Thus, we designed a bisulfite methylation assay to look for potential epimutations in the 2 known CpG islands of the *SDHC* promoter regions, CpG17 and CpG27. This analysis was performed in 4 dSDH (including GIST_21 and 3 GIST without SDHx mutations) and in 8 *KIT/PDGFRA*-mutated GIST. Hypermethylation of the *SDHC* promoter was found only in GIST_21, and this alteration was detected in heterozygosis at all CpG sites (Fig. 1c). The methylated case showed a significant 0.63-fold decrease in *SDHC* mRNA expression level (*P* = 0.0001) in comparison with the 11 methylation-negative cases (Fig. 1d).

## Discussion

Previous studies showed that all dSDH GIST are characterized by a genome-wide hypermethylation phenotype, but the downregulation of the *SDH* complex remains unexplained for those cases lacking mutations in the SDHx subunits. The case of dSDH GIST that we report here has not shown any molecular alterations in the SDHx complex nor have other pathogenetic events been detected by whole exome sequencing. Epigenetic modification of the *SDHC* promoter was the only pathogenetic event identified. Haller et al. [6] reported for the first time the presence of aberrant DNA methylation of the *SDHC* promoter in 4 patients with dSDH Carney triad–related GIST that are known to lack SDHx mutations. More recently, Killian et al. [7] performed a genome-wide methylation assay on 59 cases of dSDH GIST, selected on the basis of *SDHB*-negative immunostaining. They detected recurrent hypermethylation of the *SDHC* promoter in 94 % (15 of 16 cases) of the SDHx wild-type subgroup (ie, deficiency of the *SDHB* protein but no mutations in the coding region of any SDH subunits). This event was not confirmed outside of the *SDHC* promoter, in which there was no significant hypermethylation. In our cohort, *SDHC* epimutation was not detected in either SDHx- or *KIT*/*PDGFRA*-mutated GIST. This finding is consistent with those of previous reports [6, 7], in which *SDHC* hypermethylation was found only in dSDH GIST without SDHx mutation. Moreover, Killian et al. [7] found that the *SDHC* methylation was mutually exclusive of mutations or loss of heterozygosity events in other SDHx subunits, with the exception of hemimethylation that was associated with heterozygous mutations of the *SDHC* gene (4 of 7 cases). In particular, the authors found 4 cases with monoallelic methylation of *SDHC* in compound heterozygosity with mutation of *SDHC,* consistent with the Knudson 2-hit model, whereas 3 cases were negative for any other alterations. The case we reported, GIST_21, could be part of this latter group, also confirming that the detection of partial methylation of *SDHC* could be a marker of the complete disassembly of the SDH complex through the down-modulation of *SDHC* and the consequent degradation of the *SDHB* protein.

The presence of additional undetected mutations or INDELs in *SDHC* was not likely because the Exome sequencing probes also covered the proximal *SDHC* promoter region and the single-nucleotide polymorphism array did not detect any copy number alterations. Otherwise, due to technical limitations of the sequencing technologies, we could not exclude the presence of other alterations in *SDHC* or other SDHx subunits that could lead to the complete absence of the *SDHB* protein (e.g., a mutation in a region not sufficiently covered by exome capture or a cryptic deletion inside the *SDHC* gene). However, considering the rarity and complexity of these molecular events, the clinical implications of these differences in SDH complex deregulation are still not completely known but probably are not associated with a different clinical profile and different behavior among the dSDH GIST cases.

## Conclusion

In conclusion, herein we reported an additional case of dSDH GIST without SDHx mutation, but harboring hypermethylation in the *SDHC* promoter, thus confirming the relevance of an epigenomic event in this pathology. For this patient, no other molecular pathogenic event was detected. Our case may confirm the complexity of the molecular background of dSDH GIST by the integration of genomic and epigenomic assays using high-throughput technologies that helped to identify the specific mechanism of SDH complex inactivation underlying the loss of *SDHB* protein expression not driven by SDHx mutations in this subgroup of GIST.

## Consent

Written informed consent was obtained from the patient for publication of this Case report. A copy of the written consent is available for review by the Editor of this journal.

## Additional files

Additional file 1:
**CARE checklist.** (DOCX 1488 kb) Additional file 2:
**Materials and methods.** (DOCX 13 kb)

## Abbreviations

GIST: Gastrointestinal stromal tumors
SDH: Succinate dehydrogenase
dSDH: SDH deficient

## References

[1] Rubin BP, Heinrich MC. Genotyping and immunohistochemistry of gastrointestinal stromal tumors: an update. Semin Diagn Pathol. 2015. [Epub ahead of print]. 10.1053/j.semdp.2015.02.017 25766843

[2] Janeway KA, Kim SY, Lodish M, Nosé V, Rustin P, Gaal J (2011). Defects in succinate dehydrogenase in gastrointestinal stromal tumors lacking KIT and PDGFRA mutations. Proc Natl Acad Sci U S A.

[3] Pantaleo MA, Astolfi A, Urbini M, Nannini M, Paterini P, Indio V (2014). Analysis of all subunits, SDHA, SDHB, SDHC, SDHD, of the succinate dehydrogenase complex in KIT/PDGFRA wild-type GIST. Eur J Hum Genet.

[4] Mason EF, Hornick JL (2013). Succinate dehydrogenase deficiency is associated with decreased 5-hydroxymethylcytosine production in gastrointestinal stromal tumors: implications for mechanisms of tumorigenesis. Mod Pathol.

[5] Killian JK, Kim SY, Miettinen M, Smith C, Merino M, Tsokos M (2013). Succinate dehydrogenase mutation underlies global epigenomic divergence in gastrointestinal stromal tumor. Cancer Discov.

[6] Haller F, Moskalev EA, Faucz FR, Barthelmeß S, Wiemann S, Bieg M (2014). Aberrant DNA hypermethylation of SDHC: a novel mechanism of tumor development in Carney triad. Endocr Relat Cancer.

[7] Killian JK, Miettinen M, Walker RL, Wang Y, Zhu YJ, Waterfall JJ (2014). Recurrent epimutation of SDHC in gastrointestinal stromal tumors. Sci Transl Med.

[8] Pantaleo MA, Astolfi A, Nannini M, Ceccarelli C, Formica S, Santini D (2011). Differential expression of neural markers in KIT and PDGFRA wild-type gastrointestinal stromal tumours. Histopathology.

[9] Astolfi A, Nannini M, Pantaleo MA, Di Battista M, Heinrich MC, Santini D (2010). A molecular portrait of gastrointestinal stromal tumors: an integrative analysis of gene expression profiling and high-resolution genomic copy number. Lab Invest.

