# Supplementary material for: SDHC methylation in gastrointestinal stromal tumors (GIST): a case report
Source: BMC Med Genet. 2015 Sep 28;16:87. doi: 10.1186/s12881-015-0233-7 (PMC4587653; doi:10.1186/s12881-015-0233-7)
Supplement: Additional file 2: — Materials and methods. (DOCX 13 kb) [file 12881_2015_233_MOESM2_ESM.docx]

**Additional file 2**

**Material and Methods**

***Whole exome sequencing***

DNA was extracted from fresh frozen tissue with the QiaAmp DNA mini kit (Qiagen, Milan, Italy), following the manufacturer’s instructions. Whole exome library was prepared in accordance with the Nextera Exome Enrichment protocol (Illumina, San Diego, CA). DNA library size was checked with Agilent DNA 1000 chips on the Bioanalyzer 2100 (Agilent Technologies, Taoyuan City, Taiwan) and then quantified using picogreen assay (Life Technologies, Monza, Italy). 12pM paired-end libraries were amplified and ligated to the flowcell by bridge PCR, and sequenced at 2 × 100-bp read length using the HiScanSQ sequencer (Illumina). Reads were mapped with BWA software against the human reference genome (HG19) collected from the UCSC Genome Browser (http://www.genome.ucsc.edu/). After various quality filters, single-nucleotide variants and INDELs were identified using the MuTect algorithm. Bioinformatic mutation prediction tools (PolyPhen2 and SIFT) were used to determine pathogenicity of the emerging variations.

***Western blot***

SDHA and SDHB protein expression was evaluated by Western blot immune assay, as described elsewhere [3].

***Methylation assay***

Genomic DNA was treated with bisulfite using the Methylamp DNA modification kit (Epigentek, Farmingdale, NY), following the manufacturer’s instructions. Primers specific for amplification of bisulfite-modified DNA and targeted to 2 CpG islands located in the promoter of *SDHC* (CpG17 chr1:161283776-161284007 and CpG27 chr1:161284119-161284451) were designed using MethPrimer (http://www.urogene.org/methprimer/). Amplicons were then sequenced on both strands using the Big Dye Terminator v1.1 cycle sequencing kit (Life Technologies). Primer sequences were 5′-GAAAATAATTAGTAAATTAGTTAGGTAG-3′ and 5′-ACTAAAATCACCTCAACAACAAC-3′ for CpG27 [6], 5′-AGGTTATAATTTATGTATTTGTTTGGTTAA-3′, and 5′-TCTTTAAAAAACTCTAAAACTTCTCC-3′ for CpG17.

***Statistics***

*SDHC* gene expression levels were obtained from previously published work (GSE20710) [8]. Probe-level expression of *SDHC* between GIST_21 and 11 methylation-negative GIST samples (3 SDHA and 8 KIT/PDGFRA mutants) were compared by one sample t-test.
